# Salivary oxytocin and amygdalar alterations in functional neurological disorders

**DOI:** 10.1093/braincomms/fcae455

**Published:** 2024-12-16

**Authors:** Samantha Weber, Natascha Stoffel, Juan Ansede-Bermejo, Raquel Cruz, Álvaro Del Real Bolt, Rupert Bruckmaier, Ángel Carracedo, Selma Aybek

**Affiliations:** Department of Neurology, Psychosomatic Medicine Unit, Inselspital Bern University Hospital, University of Bern, Bern 3012, Switzerland; Department of Adult Psychiatry and Psychotherapy, University Hospital of Psychiatry Zurich, Zurich 8032, Switzerland; Department of Neurology, Psychosomatic Medicine Unit, Inselspital Bern University Hospital, University of Bern, Bern 3012, Switzerland; Faculty of Science and Medicine, University of Fribourg, Fribourg 1700, Switzerland; Graduate School of Health Science (GHS), University of Bern, Bern 3013, Switzerland; Centro Nacional de Genotipado (CEGEN), Universidade de Santiago de Compostela, Santiago de Compostela 15706, Spain; Centro Nacional de Genotipado (CEGEN), Universidade de Santiago de Compostela, Santiago de Compostela 15706, Spain; Centre for Biomedical Network Research on Rare Diseases (CIBERER), Instituto de Salud Carlos III, Madrid 28029, Spain; Instituto de Investigación Sanitaria de Santiago (IDIS), Hospital Clínico Universitario de Santiago (CHUS), Santiago de Compostela 15706, Spain; Centro Singular de Investigación en Medicina Molecular y Enfermedades Crónicas (CIMUS), Universidade de Santiago de Compostela, Santiago de Compostela 15706, Spain; Medicine and Psychiatry Department, University of Cantabria, Santander 39005, Spain; Veterinary Physiology, Vetsuisse Faculty, University of Bern, Bern 3012, Switzerland; Centro Nacional de Genotipado (CEGEN), Universidade de Santiago de Compostela, Santiago de Compostela 15706, Spain; Centre for Biomedical Network Research on Rare Diseases (CIBERER), Instituto de Salud Carlos III, Madrid 28029, Spain; Instituto de Investigación Sanitaria de Santiago (IDIS), Hospital Clínico Universitario de Santiago (CHUS), Santiago de Compostela 15706, Spain; Centro Singular de Investigación en Medicina Molecular y Enfermedades Crónicas (CIMUS), Universidade de Santiago de Compostela, Santiago de Compostela 15706, Spain; Fundación Pública Galega de Medicina Xenómica, Sistema Galego de Saúde (SERGAS), Santiago de Compostela 15706, Spain; Faculty of Science and Medicine, University of Fribourg, Fribourg 1700, Switzerland

**Keywords:** conversion disorder, epigenetics, *OXTR*, rs53576, amygdala

## Abstract

Individuals diagnosed with functional neurological disorder experience abnormal movement, gait, sensory processing or functional seizures, for which research into the pathophysiology identified psychosocial contributing factors as well as promising biomarkers. Recent pilot studies suggested that (epi-)genetic variants may act as vulnerability factors, for example, on the oxytocin pathway. This study set out to explore endogenous oxytocin hormone levels in saliva in a cohort of 59 functional neurological disorder patients and 65 healthy controls comparable in sex and age. First, we examined the association between salivary oxytocin levels with the genetic allelic variant (rs53576) of the oxytocin receptor gene (*OXTR*), its epigenetic changes indicated by methylation rates, and clinical variables—including childhood trauma. Second, due to previously reported effects of oxytocin changing the volume and functional connectivity of the amygdala, as well as the known involvement of the amygdala in the pathophysiology of functional neurological disorders, we further looked at both structural and functional imaging of the amygdala. While patients did not significantly differ from healthy control in their peripheral oxytocin levels, there was a specific interaction of *OXTR* methylation and peripheral oxytocin dependent on group: higher methylation rates correlated with higher salivary oxytocin in patients only, while this was not the case in healthy control [*F*(1109) = 8.92, *P* = 0.003, *d* = 0.541]. Moreover, patients with the AA-genotype (minor allele) of the rs53576 genetic variant of the *OXTR* gene presented with higher *OXTR* methylation levels [*F*(2106) = 10.25, *P <* 0.0001, *d* = 0.58]. Lastly, amygdalar connectivity to the hippocampus, the posterior cingulate cortex, the inferior parietal cortex and the inferior temporal cortex as well as smaller amygdalar volume were correlated to peripheral oxytocin levels in patients only [*F*(2,38) = 5.36, *P*  = 0.025,  *d* = 0.431], but not in healthy control. No significant interactions with childhood trauma were identified. Our study revealed a significant interplay between peripheral oxytocin and *OXTR* methylation in patients only, potentially influenced by genotype. One could hypothesize that higher peripheral oxytocin denotes a compensatory mechanisms for the increased methylation of the *OXTR*, which might affect amygdalar functional connectivity. These findings help to further understand underlying pathophysiological mechanisms, considering oxytocin’s involvement in functional patients and could offer a potential site of treatment for future studies.

## Teaser

Weber and Stoffel *et al*. report on 59 patients with functional neurological disorder and 65 controls, exploring salivary oxytocin, oxytocin receptor gene variants and methylation. Patients showed higher oxytocin linked to increased methylation, which may relate to changes in functional connectivity around the amygdala and may suggest new treatment avenues.

## Graphical Abstract

**Graphical Abstract:**
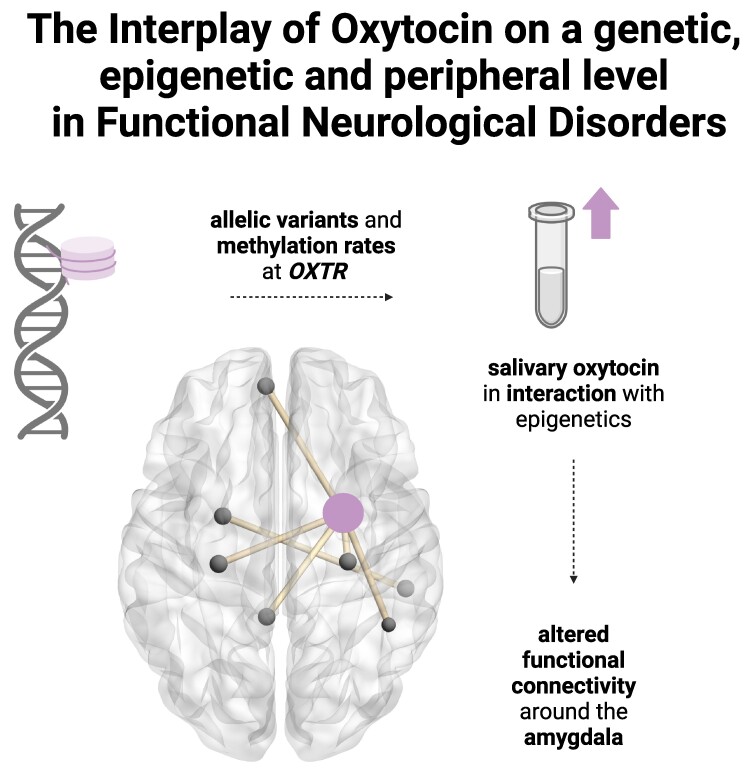

## Introduction

The pathophysiology underlying functional neurological disorder (FND) has been extensively studied.^1^ Contributing factors such as exposure to psychosocial stressors are of relevance^2^ and some biomarkers were recently identified through neuroimaging: state markers, such as a reduced resting brain metabolism in frontal regions^3^ or trait markers such as reduced amygdalar and hippocampal volumes may play a role in FND.^4^ How these contributing factors and brain changes are interrelated is still unknown, and a promising new area of research in FND is looking at the potential role of genetic and epigenetic changes.^5,6^ Preliminary results identified increased oxytocin receptor gene (*OXTR*) methylation in a small cohort of patients with FND,^7^ suggesting a potential involvement of the oxytocinergic system in the pathophysiology of FND. Indeed, the hormone oxytocin (OXT) plays an important role in many physical and psychological health-related processes in humans, including autonomic, emotional and behavioral changes.^8^ Current hypotheses postulate that OXT has an inhibitory effect on the corticotrophin-releasing factor (CRF) gene expression and may therefore help to prevent chronic manifestation of stress-symptoms,^8,9^ which is of high interest in stress-related neuropsychiatric disorders such as FND.^10^

Endogenous OXT is released from the posterior pituitary and might buffer the stress response by decreasing activity in the hypothalamus-pituitary-adrenal (HPA) axis.^9^ Previous studies showed that salivary OXT levels not only correlated with parasympathetic activity (as measured using heart rate variability)^11,12^ but also exerted a regulatory effect on the HPA axis,^13,14^ through neuroimmunological processes.^15,16^ While OXT’s involvement has been discussed in disorders such as anxiety, borderline personality, schizophrenia and autism spectrum disorder,^17,18^ only one study looked at the correlation of peripheral levels in OXT and clinical variables of FND. They discussed the interaction of lower blood OXT being associated with a history of childhood trauma in patients with FND.^19^ Having endured an early life threat exposure appears to contribute to general psychopathology, particularly in subjects carrying the A allele of the rs53576 single nucleotide polymorphism (SNP) of the *OXTR* gene.^20,21^ Other studies support the finding that being a carrier of this allele can contribute to depressive symptomatology^22-25^ or increased vulnerability in the context of stress.^26^

Further, epigenetic fine tuning of the *OXTR* is important as the effect of endogenous OXT depends on the *OXTR* methylation levels.^8^ In other words, in order for OXT to bind to its receptor, the *OXTR* must be expressed, whereas higher methylation rates can lead to a reduced expression.^27-29^  *OXTR* expression was found to be upregulated (i.e. increased expression rates) in A-allele carriers, as well as survivors of childhood abuse, indicating a genetic but also psychosocial involvement in the regulation of *OXTR*.^30^

On the other hand, methylation levels were also found to be higher in children exposed to maltreatment and correlated with smaller orbitofrontal cortex (OFC) volumes.^31^ A systematic review focusing on the contribution of *OXTR* methylation to psychopathologies showed evidence that indeed higher rates of methylation are linked to abnormal social behaviours, while only two studies also looked at the correlation between *OXTR* methylation and peripheral OXT levels.^28,32^ So far, a negative correlation between peripheral (blood-derived) levels of OXT and *OXTR* methylation rates was found in adolescents, which interacted with their callus-unemotional traits.^28^ Further, the correlation of *OXTR* methylation and peripheral (blood-derived) OXT levels were found to be negative for males, while a positive correlation was found for female psychotic patients.^32^ Additional sex-specific associations of *OXTR* methylation show that females higher *OXTR* methylation levels were correlated with higher early life adversity.^33^

On a neurobiological level, peripheral OXT in plasma seems to correlate with the right amygdala both structurally and functionally, as higher endogenous levels were associated with reduced central or lateral amygdalar volume^34,35^ and reduced activation following aversive stimuli.^34^ Animal studies confirmed that the central amygdala is rich in OXT receptors and may be selectively affected by changes in the oxytocin system^36^ and that OXT receptors in the central amygdala modulate the fear response.^35,37^ This is remarkable as more nuanced brain imaging studies in females with functional seizures have shown a reduced volume of right lateral amygdala, but an enlarged volume of the right central amygdala compared with controls.^38^ Also, peripheral levels of salivary OXT correlated with low interregional functional coupling between the amygdala and hippocampus,^39^ while enhanced strength of amygdala-hippocampal functional connectivity (FC) has been associated with emotional abuse.^40^ The activity and reactivity of the amygdala seem highly dependent on OXT, especially considering context and valence.^41-44^

Studying the contribution of OXT as an endogenous hormone, along with its genetic and epigenetic contribution to a stress-related disorder like FND is highly relevant,^45^ eventhough genetic and epigenetic studies require large cohorts.^46^ Thus, in this study, we aimed to look at the peripheral levels of salivary OXT, taking into account the genotype variant at rs53576 SNP of the *OXTR* gene, the *OXTR*’s methylation rates and neuroimaging data (both structural and resting-state FC) in a well-characterized population of patients with mixed FND symptoms compared with healthy controls (HCs). We hypothesized that patients with FND would show lower levels of salivary OXT and higher *OXTR* methylation interacting with the occurrence of childhood trauma. Further, we hypothesized that peripheral, epigenetic and genetic markers of the oxytocin system would correlate negatively with amygdalar volume and connectivity.

## Materials and methods

### Participants

The study was carried out at the University Hospital Inselspital Bern, Switzerland. For the exact study design and procedure on the assessment of demographic and clinical characteristics, we kindly refer the reader to our previous work.^4,47^ Genetic data of the same cohort have previously been published elsewhere.^48^ The study was approved by the local ethics committee of the Canton Bern (SNCTP000002289) and conducted according to the Declaration of Helsinki. Written informed consent was provided by all subjects. All participants underwent a magnetic resonance imaging (MRI) scan, provided blood and saliva samples and completed questionnaires on depression [Beck’s Depression Inventory (BDI^49^)], state and trait anxiety [State-Trait Anxiety Inventory (STAI^50^)] and childhood trauma [Childhood Trauma Questionnaire (CTQ^51^)].

To determine the sample size of required participants to obtain results that we can reliably interpret in its significance, we conducted two independent sample size calculations based on previous findings in FND. For our main outcome, the peripheral levels of OXT mediated through experience of childhood trauma, we based our sample size calculation on the results of Örnek *et al*.^19^ (*N*_Childhood Trauma YES_ = 14, *N*_Childhood Trama NO_ = 13, effect size = 0.682). This indicated a required sample size of *N* = 96, i.e. 48 per group, when using an alpha error score of 0.05, and a power set at 0.95 in an independent *t*-test. For the secondary variable of interest, the methylation rates of *OXTR*, we based our calculation on preliminary results from Apazoglou *et al*.^7^ (*N*_FND_ = 16, *N*_HC_ = 15, effect size = 0.98), in which overall methylation rates (the sum of the two sites) were used. This indicated a required sample size of *N* = 48, split in two groups, when using an alpha error score of 0.05, and power set at 0.95 in an independent *t*-test.

### Salivary oxytocin

Salivary OXT measurements are physiologically validated^52^ eventhough the precise mechanisms behind OXT entering peripheral saliva have not been entirely understood. Nonetheless, saliva presents arguably the easiest and most reliable measurement option, even without extraction of potentially interfering molecules^53,54^ as compared with plasma, there is high protein interference^55^ as well as high dependency on the exact methods.^54^ Moreover, salivary OXT levels correlate better with central OXT in the cerebrospinal fluid compared with plasma measurements, thus it probably mirrors more likely the OXT active in the brain.^56-58^ To account for some of the potential timely variance and to provide sufficient saliva volume per subject, saliva samples were collected from each participant before entering the MRI scanner as well as after completion of the MRI scan. Salivette collection devices (Sarstedt, Germany) were used, and the two samples per subject were pooled before analysis. Salivary OXT was measured using an ultra-sensitive ELISA by Enzo Life Sciences, with a sensitivity of 15 pg/mL OXT (https://www.enzolifesciences.com/ADI-901-153A/oxytocin-elisa-kit/), which is one of the most commonly used assay kit for saliva when looking at OXT-related studies in a recent the meta-analysis.^59^ Before measuring individual patient samples, the ELISA kit was validated using pooled saliva samples spiked prior to the extraction steps with oxytocin standards to achieve concentrations of 10, 20, 50 and 100 pg/mL, in addition to the concentration in the native pool. Recovery ranged from 110% to 120%, as predicted for saliva samples in the kit manual. Because native concentrations were very low and often below the detection limit, we performed a 4-fold up-concentration by using 1 mL of saliva for the extraction to be reconstituted in 250 µL of assay buffer.

### DNA samples

A total of 15 mL of whole blood using two 7.5 mL EDTA S-Monovette tubes (Sarstedt, Nümbrecht, Germany) was withdrawn from each participant and frozen at −20°C. DNA was extracted according to the manufacturer’s protocol using the QIAmp DNA Blood kit (Qiagen, Hilden, Germany). In each sample, DNA concentration was quantified using Quant-it dsDNA Broad-Range Assay Kit (Invitrogen, Thermo Fisher Scientific, MA, USA) according to the manufacturer’s protocol and further normalized to 60 µL with a double-stranded DNA concentration of 20 ng/µL. Genotyping and methylation analysis were conducted at the Spanish National Center for Genotyping (CeGEN, Santiago de Compostela, Spain).

### Selection of regions and primer design

CpG islands of the *OXTR* genes were selected based on previous findings in patients with FND, in which increased methylation was identified compared with HC.^7^ Furthermore, all participants were genotyped for the rs53576 SNP of the *OXTR* gene. The assay design for the methylation analysis was created using EpiDesigner software (https://www.epidesigner.com/). Specificity of the design was tested using an *in silico* assay prediction performed in *R* statistical software (*MassArray* package), and primer design was tested using Primer Design and Search Tool available at http://bisearch.enzim.hu/.

### Genotyping analysis

DNA samples were genotyped using iPLEX Assay^60^ followed by mass spectrometry analysis using the MassARRAY System (Agena Bioscience, San Diego, CA, USA). The analysis included the following steps (i) PCR amplification, (ii) Shrimp Alkaline Phosphatase (SAP) treatment to remove unincorporated nucleotides and (iii) single base extension (SBE) to create nucleotide-mass-specific products. Processed samples were then dispensed on a 384-element SpectroCHIP bioarray and further analyzed using the matrix-assisted laser desorption/ionization time-of-flight (MALDI-TOF) technique within the MassARRAY Workstation. The genotyping (rs53576 of *OXTR* gene) reactions were performed using the following primers: F: TGGAAAGGAAAGGTGTACGG; R: GTAGAATGAGCTTCCCAGCC; SBE primer: TTTCTGTGGGACTGAGGA. The forward and reverse primer sequences contained all the following preceding TAG sequence ACGTTGGATG. Primers were designed using the MassARRAY Assay Design Software.

### Methylation analysis

Quantitative DNA methylation analysis was performed using EpiTYPER^61^ within the MassARRAY System, including bisulfite treatment (EZ-96 DNA Methylation MagPrep kit, Zymo Research), which converts non-methylated Cytosine (C) into Uracil (U), and a subsequent PCR amplification using 7T-promoter tags. Melting time was adjusted according to each primer. The C to U transformation causes a methylation-dependent sequence variation from C to T in the PCR amplification products. Samples further underwent SAP treatment. Ultimately, RNA transcription was performed on the reverse strand, followed by a *U*-specific cleavage. Using MALDI-TOF, mass spectrometry analysis produced a methylation-dependent signal pattern corresponding to the mass difference due to the introduced sequence variation. All samples were analyzed in duplicates. Samples with inconsistent results were reanalyzed. In all steps of the analysis samples, were processed in a randomized order to avoid a group bias. The same methylation sites were selected as in Apazoglou *et al*.^7^ namely −944 and −934 (hg19, chro3:8 810 729-8 810 845).

### Neuroimaging

All subjects underwent an anatomical MRI scan using a sagittal-oriented T1-weighted 3D-MPRAGE sequence (TR = 2330 ms, TE = 3.03 ms, TI = 1100 ms, matrix 256 × 256, FOV=256 mm × 256 mm, flip angle = 8°, resolution = 1 mm^3^ isotropic, TA = 5:27 min).^62^ Additionally, resting-state functional imaging data were acquired using a whole-brain interleaved multi-slice BOLD echo-planar-imaging (EPI) sequence (TR = 1300 ms; TE = 37 ms, ﬂip angle = 52°, FOV = 230 mm, voxel size = 2.2 mm^3^ isotropic, TA = 6:39 min, for a total of 300 functional volumes). Imaging data were pre-processed using SPM12 (https://www.fil.ion.ucl.ac.uk/spm-statistical-parametric-mapping/) in MATLAB (R2017b, MathWork Inc., Natick, MA, USA). Functional images were realigned and co-registered to the anatomical T1 image. They were subsequently detrended and covariates of no interest were regressed out (including constant, linear and quadratic trends, average white matter/cerebrospinal fluid time courses, motion artefacts and global signal). Functional volumes were filtered using a high-pass filter at 0.01 Hz. Lastly, functional images were normalized to MNI standard space and smoothed using a spatial Gaussian kernel of 5 mm full width at half maximum.^63,64^

### Statistical analyses

Statistical analyses were performed using *R* software (version 4.2.0.) and MATLAB (R2017b, MathWorks Inc., Natick, MA, USA). Data were tested for normality using Shapiro–Wilk test. Normally distributed demographic/clinical data were analyzed using a two-sample *t*-test, the Wilcoxon rank sum test was used. When applicable, data were corrected for multiple comparisons using false discovery rate (FDR). Alpha-level was set at *P* < 0.05 to determine significance. Cohen’s *d* reports the effect size calculated for analyses of variances.

#### Salivary oxytocin analysis

Group differences in salivary OXT data were calculated using an ANOVA on the fitted data using a linear model, corrected for age, sex, psychotropic medication (dichotomized yes/no), BDI, STAI-S, menstrual cycle, hormonal contraception and menopause (for details on selection of covariates, see Supplementary material). For the menstrual cycle, the exact day of the cycle was calculated for menstruating women (between 0 and 35 days) to account for the specific fluctuation through the cycle. A meta-analysis summarized multiple studies that tracked intra-individual OXT values across the cycles and showed a variability across days that is best tracked as a continuous variable.^65^ We further decided to set a corresponding value for those that do not have a natural cycle, according to data-guided values^65,66^: Post-menopausal women as well as male participants were set at day = 5 of the cycle as this corresponds to the standardized zero value from the meta-analysis investigating OXT-measurement across women’s cycle. Please note that this decision was made by the authors based on the existing literature and not a standardized method. There has unfortunately not yet been a standardized method that still accounts for the variability across different subgroups, while it is agreed upon that it is important to both include different subgroups and account for these covariates adequately.^65,67^ We thus decided to allow the menstrual cycle to remain a continuous variable representing the dynamics across the cycle, while adding the subpopulation without the menstrual cycle–based fluctuation into the same variable.^65^ Additionally, models including an interaction term with childhood trauma (CTQ total score) as well as each individual subscore were implemented.

#### Genotyping analysis

Genotype call rate, minor allele frequency and Hardy–Weinberg equilibrium using the χ^2^ test were calculated as quality control procedures. For sub-analyses, participants were stratified into their corresponding genotype of the rs53576 of the *OXTR* gene (GG versus GA versus AA).

#### Methylation analysis

Mean methylation rates were computed by averaging the methylation of the individual amplicons of the 2 CpG sites tested. Group differences in methylation data were calculated using an ANOVA on the fitted data using a linear model, corrected for age, sex, psychotropic medication, BDI, STAI-S, menstrual cycle, contraception and menopause. Additionally, models including an interaction term with childhood trauma (CTQ total score) as well as each individual subscore (emotional abuse, emotional neglect, physical abuse, physical neglect and sexual abuse) were implemented. Lastly, a model with an interaction term with sex was implemented based on prior evidence that sex differences might occur with regards to methylation^32^ (Supplementary Fig. 4).

#### Interaction between oxytocin, *OXTR* methylation and genotype

We investigated group differences in salivary OXT, including an *OXTR* methylation interaction term in the model in patients with FND and HC, using an ANOVA on the fitted data using a linear model with age, sex, psychotropic medication, BDI, STAI-S, menstrual cycle, contraception and menopause as covariates of no interest. Secondly, the effect of genotype (rs53576 of the *OXTR* gene) on *OXTR* methylation was investigated.

#### Oxytocin and structural brain alterations

Based on the *a priori* hypothesis that the amygdala would be the key region of interest from its involvement in the oxytocinergic system^34,35,39,40,68,69^ as well as neurological or psychiatric involvement,^17,18^ we investigated the association between salivary OXT and the amygdalar brain volume. Total intracranial volume, age, sex, psychotropic medication, BDI, STAI-S, menstrual cycle, hormonal contraception and menopause were used as covariates of no interest. Anatomical images were pre-processed using the Computational Anatomy Toolbox (CAT12—http://www.neuro.uni-jena.de/cat/) within SPM12 (https://www.fil.ion.ucl.ac.uk/spm/software/spm12/) according to standard voxel-based morphometry (VBM) procedures.^70,71^ These steps include the application of a spatial adaptive non-local means denoising filter,^72^ bias correction, affine registration and subsequent segmentation.^73^ Furthermore, an adaptive maximum a posteriori (AMAP) segmentation^74^ step was applied, and fractional content of each tissue type was estimated using a partial volume estimation.^75^ Images were normalized using DARTEL registration^76^ and further smoothed using an isotropic FWHM kernel of 8 mm. VBM with CAT12 was chosen for its suitability in accurately analyzing subcortical volumes, like the amygdala, due to its advanced segmentation and normalization capabilities, as well as its computational efficiency.^71^ Lastly, subject-wise estimates of mean amygdalar volume were extracted.

#### Oxytocin and resting-state functional connectivity

Seed-to-whole-brain resting-state FC using the amygdala as the seed region was calculated according to standard procedure.^63,64,77^ As such, the functional images were parcellated into the 90 cortical and subcortical regions of interest according to the automatic anatomic labelling atlas. The region-averaged time courses were extracted, and FC was computed using Pearson’s correlation coefficient between the time series of the seed with each of the regions. The correlation coefficients were further *z*-scored using Fisher *z* transformation. First, significant differences in FC between patients and controls were assessed using two-tailed multiple *t*-tests, corrected using FDR at a significance threshold of *P* < *α,* where alpha level (*α*) was set to 0.05. Second, the correlation between salivary OXT and amygdala-to-whole-brain FC was evaluated by computing connection-wise Pearson’s correlation coefficient with salivary OXT data. Significance threshold was set at *P* < *α,* where alpha level (*α*) was set to 0.05.

## Results

### Clinical and demographic characteristics

OXT was measured in saliva samples that have been collected within the framework of previous work.^4,47^ Since the determination of OXT levels in saliva requires sufficient volume, we were able to determine the salivary OXT levels in only 59 patients with FND and 65 HC (out of the total 86 patients with FND and 76 HC). But based on our two sample size calculations, a sample of *N*_total_ = 124 still seems appropriate to claim the presented finding to be statistically valid, with *N*_FND_ = 59 (76.3% female) and *N*_HC_ = 65 (73.8% female). Compared with HC, patients with FND scored significantly higher in depression, state- and trait- anxiety levels, as well as total CTQ score and CTQ emotional neglect Table 1.

**Table 1:** Demographic and clinical data

	FND (*N* = 59)	HC (*N* = 65)	Statistics
Age, mean (SD), years, (range)	37.7 (14.2), (17–77)	33.1 (10.9), [18–62]	*Z* = −1.93, *P* = 0.053
Sex (females/males)	45/14	48/17	*Χ* ^2^(1) = 0.01, *P* = 0.92
Hormonal contraception (yes/no)	26/19	27/15	*Χ* ^2^(1) = 2.39, *P* = 0.30
Menopause (yes/no)	12/33	10/38	*Χ* ^2^(1) = 0.54, *P* = 0.77
Menstrual cycle	14 anovulation 7 follicular 16 luteal 2 menstruation 6 ovulation	11 anovulation 3 follicular 30 luteal 1 menstruation 3 ovulation	Two-tailed *P* = 0.17
Disease severity (CGI, median, quantile)	3 (1–4)	*NA*	
Duration of illness (in months)	60.12 (71.46)	*NA*	
Symptom type^a^	31 sensorimotor 18 gait disorder 15 tremor 8 myoclonus 10 PNES 3 dystonia 3 PPPD 3 speech disorder 1 functional deafness	*NA*	
ICD-10 classification^b^	44 F44.4 5 F44.5 22 F44.6 6 F44.7	*NA*	
Psychotropic medication	8 benzodiazepines 17 antidepressants 3 neuroleptics 7 antiepileptics 4 opioids	0/76	
**BDI score, mean (SD)**	**15.1 (10.91)**	**4.6 (6.4)**	*Z* = −6.78, *P* < 0.0001***
**STAI-S score, mean (SD)**	**36.1 (11.24)**	**32.2 (7.5)**	*Z* = −1.97, *P* = 0.049*
**STAI-T score, mean (SD)**	**45.5 (13.2)**	**33.8 (7.3)**	*Z* = −5.20, *P* < 0.0001***
**CTQ total, mean (SD)**	**43.7 (18.6)**	**36.8 (14.3)**	*Z* = −2.30, *P* = 0.02*
**Emotional neglect**	**11.3 (5.4)**	**9.0 (4.3)**	*Z* = −2.55, *P* = 0.01*
Emotional abuse	10.3 (5.6)	8.4 (4.2)	*P* = 0.1
Physical neglect	7.6 (3.3)	6.9 (3.0)	*P* = 0.09
Physical abuse	7.4 (4.3)	5.9 (2.1)	*P* = 0.09
Sexual abuse	7.1 (4.1)	6.6 (3.7)	*P* = 0.09

BDI, Beck’s Depression Inventory; CGI, Clinical Global Impression Score; CTQ, Childhood Trauma Questionnaire; FND, Functional Neurological Disorder; HC, Healthy Controls; ICD, International Classification of Diseases; STAI, State-Trait Anxiety Inventory.

^a^Patients can present with several symptom types.

^b^Diagnosis of mixed FND (F44.7) was given when F44.4, F44.5 and F44.6 was present.

Significance level: ***P < 0.001, *P < 0.05. Significant group differences are shown in bold.

### Salivary oxytocin

Corrected for age, sex, psychotropic medication, BDI, STAI-S, menstrual cycle, hormonal contraception and menopause, patients with FND (mean ± SD: 7.93 pg/mL ± 3.76 pg/mL) did not significantly differ in their salivary OXT levels, compared with HC (mean ± SD: 7.16 pg/mL ± 3.61 pg/mL), *P* = 0.25. No interaction with total score on childhood trauma nor any of the CTQ subscores was identified.

### Genotyping

There were *N* = 12 patients with FND (10% of the full population, 20% of the FND population) and *N* = 6 HC (5% of the full population, 9% of the HC population) with the AA genotype, while there were *N* = 47 patients with FND (38% of the full population, 80% of the FND population) and *N* = 59 HC (47% of the full population, 91% of the HC population) with the GG or GA genotype, Table 2. As such, a significant association between FND and the AA genotype of the rs53576 of the *OXTR* gene was identified [recessive model: OR = 3.96, CI = (1.13–13.93), *P* = 0.03]. Age, sex, BDI, STAI-T and total CTQ score were used as covariates.

**Table 2:** Association analysis between SNP and FND

Gene	SNP ID	Model	Genotype	Cases			
				FND	Controls	OR (95% CI)	*P*-value
*OXTR*	rs53576	Recessive	G/G-G/A A/A	47 12	59 6	1.00 3.96 (1.13–13.93)	0.028*

*OXTR*, oxytocin receptor.

Significance code: *P < 0.05.

### Methylation

Methylation levels could not be determined in two patients with FND and one HC. *OXTR* mean methylation levels in patients with FND (mean methylation ± SD: 39.8% ± 4.1%) did not significantly differ from mean methylation levels in HC (mean methylation ± SD: 38.9% ± 3.6%), *P* = 0.17. There was also no sex effect detected (see Supplementary Fig. 4).

### Interaction between oxytocin, *OXTR* methylation and genotype

**Figure 1:**
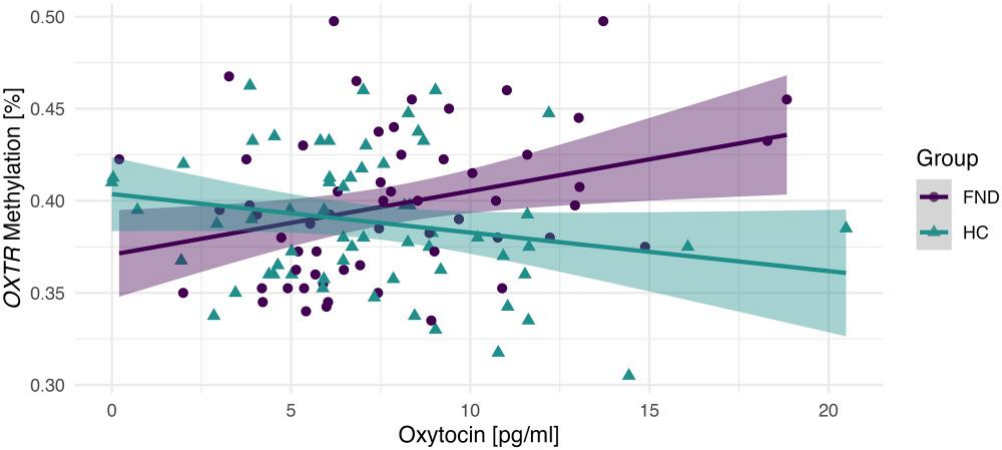
**Interaction between peripheral oxytocin levels and OXTR methylation.** Scatter plot illustrating the association between oxytocin levels (pg/mL) and OXTR methylation (%) in *N* = 59 FND patients and *N* = 65 HC. Data points represent individual participants, with their position on the *x*-axis corresponding to their oxytocin levels (pg/mL), and the *y*-axis corresponding to their OXTR methylation level (%). In patients with FND, higher peripheral oxytocin levels correlated with increased OXTR methylation [*F*(1109) = 8.92, *P* = 0.003, *d* = 0.541], whereas an inverse (but not significant) correlation was identified in HC.

A significant interaction effect between group and *OXTR* methylation on salivary OXT was identified [*F*(1109) = 8.92, *P* = 0.003, *d* = 0.543]. This suggests that the groups differ in the way *OXTR* methylation and salivary OXT levels interact. Namely, higher salivary OXT levels in FND were associated with higher *OXTR* methylation levels, while the opposite was found in HC, Fig. 1. No interaction with childhood trauma was found (see Supplementary Figs. 2 and 3).

**Figure 2:**
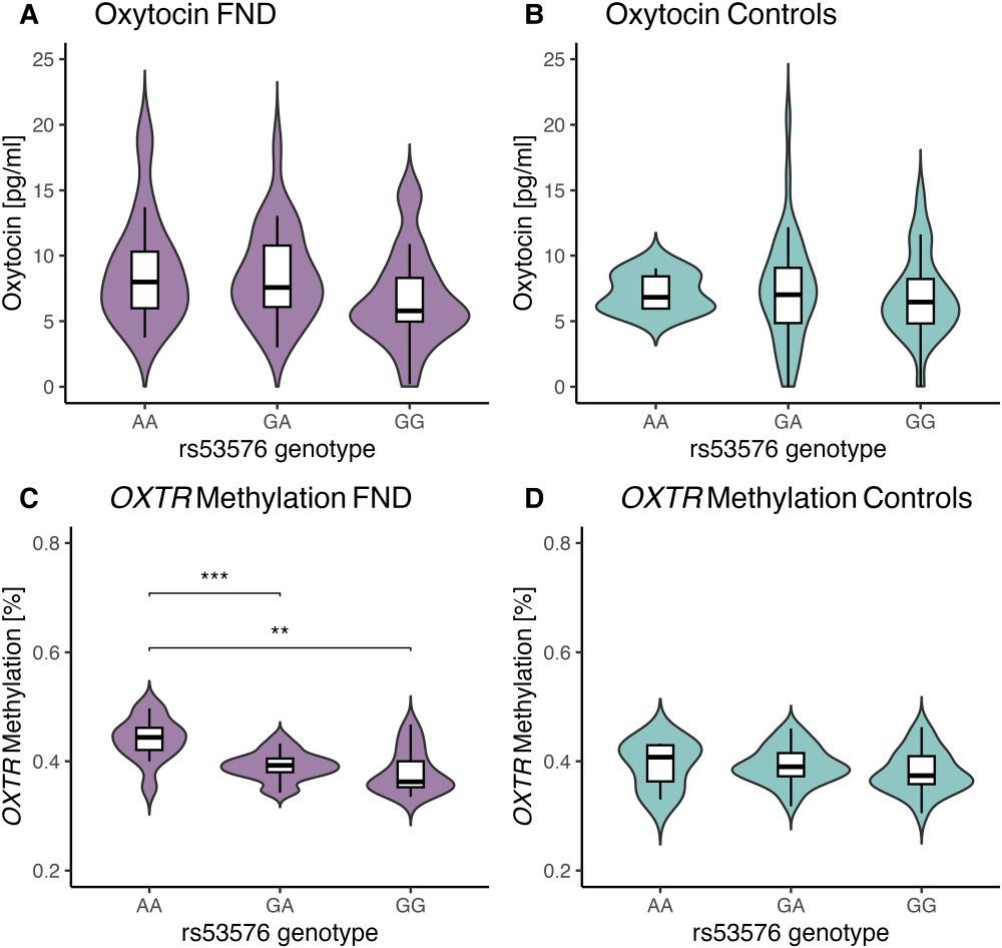
**Oxytocin and OXTR methylation according to genotype.** Violin plot representing distribution of raw salivary/peripheral oxytocin levels in (**A**) patients with FND (*N* = 59) and (**B**) HC (*N* = 65), as well asOXTR methylation levels in (**C**) patients with FND (*N* = 59) and (**D**) HC (*N* = 65). Boxplots indicate median and interquartile range. A significant main [*F*(2106) = 10.25, *P* < 0.0001, *d* = 0.583] effect of genotype and interaction [*F*(2106) = 3.27, *P* = 0.042, *d* = 0.329) effect between group and genotype on OXTR methylation was found in FND patients (**C**) using an ANOVA on the fitted data using a linear model indicating higher methylation levels in AA carriers compared with GA (*P* < 0.0001) and GG (*P* < 0.0001) carriers. Significance codes: ***P < 0.001, **P < 0.01.

Furthermore, there was a significant main effect of genotype on *OXTR* methylation [higher methylation in subjects with the AA variants *F*(2106) = 10.25, *P <* 0.0001, *d* = 0.583], together with a significant interaction effect between group and genotype on *OXTR* methylation [*F*(2106) *=* 3.27, *P* = 0.042, *d* = 0.329]. In patients only, *post hoc* Tukey’s HSD test revealed that methylation rates were significantly higher in AA carriers, compared with GA (*P* < 0.0001) and GG (*P* < 0.0001) among patients with FND, which was not the case in HC, Fig. 2. No interactions with childhood trauma nor sex were found.

### Oxytocin and structural brain alterations

In patients with FND but not in HC, higher levels of salivary OXT were associated with smaller right amygdalar brain volume [*F*(2,38) = 5.36, *P*  = 0.025,  *d* = 0.421], similar to previous studies.^34,35^ No interactions with genotype nor childhood trauma were identified in both groups.

### Oxytocin and resting-state functional connectivity

Functional imaging data from four patients and one HC had to be excluded due to too high motion artifacts, and data from one patient due to a history of drug abuse, leading to a sample of 54 patients and 64 HC that were included in the subsequent analysis. Whole-brain resting-state FC analysis, independent of OXT, can be found in Supplementary Fig. 1.

**Figure 3:**
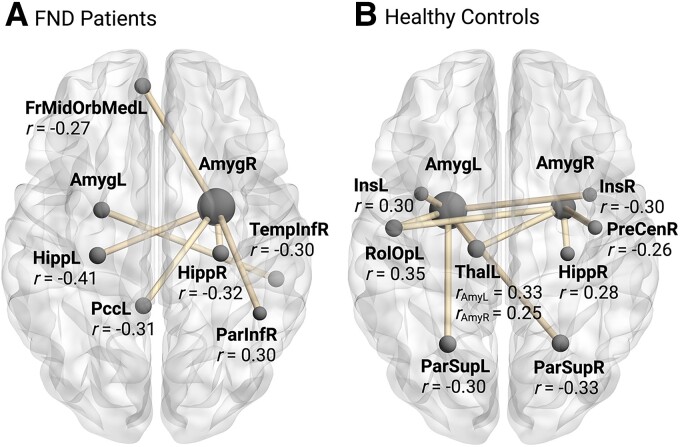
**Correlation between amygdala-to-whole-brain FC and peripheral oxytocin levels in (A) patients with FND and (B) HC.** The correlation between salivary oxytocin levels and amygdala-to-whole-brain FC was evaluated using Pearson’s correlation coefficient in *N* = 54 patients with FND compared with *N* = 64 HC. Significant correlations [with alpha level (α) set to 0.05] are shown with their associated Pearson’s correlation coefficient (*r*). FND, functional neurological disorder; FrMidOrbMedL, left frontal medial orbital cortex; AmygL/R, left/right amygdala; HippL/R, left/right hippocampus; PccL, left posterior cingulate cortex; ParInfR, right inferior parietal cortex; TempInfR, right inferior temporal cortex; InsL/R, left/right insula; r, Person’s correlation coefficient; RolOpL, left rolandic operculum; ThalL, left thalamus; ParSupL/R, left/right superior parietal cortex; PreCenR, right precentral gyrus. Figure was created using BrainNet Viewer https://www.nitrc.org/projects/bnv/.

In patients with FND, salivary OXT levels correlated with FC between the right amygdala and (i) the left hippocampus (*r* = −0.41, *P* = 0.002), (ii) the right hippocampus (*r* = −0.32, *P* = 0.02), (iii) the left posterior cingular cortex (*r* = −0.31, *P* = 0.02), (iv) right inferior parietal cortex (*r* = 0.30, *P* = 0.03) and (v) the left frontal medial orbital cortex (*r* = −0.27, *P* = 0.04), as well as between the left amygdala and the right inferior temporal cortex (*r* = −0.30, *P* = 0.03), Fig. 3A.

In HC, salivary OXT levels correlated with FC between the left amygdala and (i) the left rolandic operculum (*r* = 0.35, *P* = 0.005), (ii) the left insula (*r* = 0.30, *P* = 0.02), (iii) the right insula (*r* = −0.30, *P* = 0.01), (iv) the left superior parietal cortex (*r* = −0.30, *P* = 0.01), (v) the right superior parietal cortex (*r* = −0.33, *P* = 0.008) and (vi) the left thalamus (*r* = 0.33, *P* = 0.006), as well as between the right amygdala and (i) the right precentral gyrus (*r* = 0.26, *P* = 0.04), (ii) the right hippocampus (*r* = 0.28, *P* = 0.02) and (iii) the left thalamus (*r* = 0.25, *P* = 0.04), Fig. 3B.

To further investigate our observed correlation between OXT and brain connectivity, we conducted an in-depth FC analysis independent of the correlation with measured salivary OXT (Supplementary material).

## Discussion

This study set out to explore endogenous oxytocin hormone levels in saliva in a cohort of 59 patients with FND and 65 HC for which no significant group differences were identified. However, when investigating salivary OXT levels in relation to *OXTR* methylation and *OXTR* genotype in patients with FND compared with HC, we identified that higher salivary OXT levels in patients with FND were associated with higher mean methylation levels, while this was opposite to findings in HC.^7^ Unlike previous pilot data on FND, which found increased overall sum methylation rates in a very small sample of 15 patients with FND (mean methylation ± SD: 68.1% ± 4.3%) and 16 HC (mean methylation ± SD: 62.5% ± 6.8%),^7^ our study did not identify a difference in mean *OXTR* methylation rates between FND and HC. Although, as we found higher mean methylation rates in AA-carriers in our sample, a different genotypic distribution in Apazoglou *et al.*’s^7^ sample or the fact of using the sum of the methylation rates across sites rather than the mean across the two sites may explain their reported finding.

Genetically, we found that patients with FND with the AA-alleles of the rs53576 had significantly higher mean methylation levels of the *OXTR* in comparison to GA and GG carriers, which was previously found in older depressive women.^22^ In patients with FND but not in HC, smaller amygdalar volume was associated with higher salivary OXT levels. Further, salivary OXT levels correlated negatively with amygdalar FC with regions such as the hippocampus, the posterior cingulate cortex and the inferior temporal cortex. In HC on the other hand, salivary OXT levels correlated also with amygdalar FC with regions such as the insula, the superior parietal cortex, the precentral gyrus and the thalamus.

Finally, we did not find an interaction between childhood trauma and salivary OXT levels. However, another study on FND reported such an interaction using blood measures of peripheral OXT, although their results did not meet the significance threshold of *P*-value <0.05 either.^19^ Potential variances in OXT’s correlation could though also derive from differences used in the exact sampling method, as different bodily fluids might not represent the same aspect of the OXT system.^56-58^

### Interaction between oxytocin, *OXTR* methylation and genotype

Our data revealed a significant between-group interaction with regard to salivary OXT and the *OXTR* methylation (i.e. the higher the methylation, the lesser the expression of the OXTR to which released OXT can bind), which is of high interest. In HC, higher *OXTR* methylation levels were associated with reduced salivary OXT, while the opposite was found in patients with FND. So far, a previous study with psychotic patients reported an interaction of peripheral OXT measured in blood and the *OXTR* methylation (which they present in their supplements), for which a sex-specific effect was identified.^32^ Interestingly, for female psychotic patients, a positive correlation between OXT and *OXTR* methylation was found,^32^ similar to our patient sample. On the contrary, a negative correlation between OXT and *OXTR* methylation was found in male psychotic patients,^32^ which is similar to our results in HC. This suggests that sex might play a crucial factor in these interactions, and potentially also in FND as a disorder that occurs dominantly in the female sex.^78^ Despite that, we did not find a significant interaction with childhood trauma in our study, a potential sex effect could drive a differential mechanism between childhood trauma and the oxytonergic system, making females arguably more sensitive to the impact of childhood trauma^33^ via enhanced *OXTR* methylation. Particularly, childhood maltreatment in the form of emotional neglect was considered a contributing factor for FND.^4^

Further insights arise from studies on social anxiety, a frequent comorbidity in FND.^78^ Anxiety has been associated with decreased levels of *OXTR* methylation,^79^ whereby higher anxiety has been associated with lower peripheral OXT.^80^ Conclusively, lower levels of peripheral OXT have been observed when accompanied by an increased ocytocin receptor availability for this disorder. In our sample, state anxiety derived from the STAI questionnaire was neither associated with *OXTR* methylation rates nor salivary OXT measurements, neither in HC nor in FND patients. However, in FND, there appears to be a specific pattern where OXT and higher *OXTR* methylation interact differently and may be disorder specific. Systematic reviews on *OXTR* methylation indeed concluded that methylation would facilitate flexibility in the regulation of the oxytocin system in various environments and contexts and that a differential methylation pattern could be interpreted as a contributing factor.^27,81^ Thus, in FND, the biological mechanisms of *OXTR* and OXT interaction might not exert the same protective role as what has been found in HC^8^ or which has been discussed as a compensatory regulation for anxiety.^79^

Additionally to our results on salivary OXT and *OXTR* methylation, we could show that *OXTR* methylation is the highest in patients with FND with the AA-genotype on the rs53576 SNP of the *OXTR* gene, which could be considered a contributing genetic factor for FND, Fig. 2C, which has been discussed before in depressive women.^22^ The sensitivity of 20% (12 AA-carriers out of 59 patients with FND) and the positive predictive value of 67% (12 AA-carriers being patients with FND from the 18 AA-carriers of the full population) highlight this difference across group level, while causative factors still need to be explored. Research on the interaction between the three levels of *OXTR* genotype, *OXTR* methylation and peripheral OXT is sparse. Taken together, these results highlight the potential altered biological mechanisms underlying FND that include the interaction with epigenetic and peripheral changes in the oxytocinergic system.

### Structural and functional alterations

Structural and functional alterations around the amygdala have previously been identified in FND,^4,5,38,82-86^ while also being an area dependent on the oxytocinergic system,^39,44,87-91^ but not yet been studied in its interaction. In our study, amygdalar volume was significantly associated with salivary OXT in FND, with higher OXT levels corresponding to smaller amygdalar volumes. Literature so far has presented that plasma levels of OXT correlated with reduced amygdalar volume as well as with reduced activity following aversive stimuli in HC.^34,35^ Thus, OXT might exert a protective effect against morphological changes through the inverse correlation to the amygdala’s volume that has previously been discussed within the pathogenesis of anxiety.^34,35^ There are also different findings of amygdalar volume in FND independent of the correlation with OXT, which seems to depend both on the analysis (e.g. whole amygdala or more nuanced subregions) and the exact population (e.g. different subtypes of FND included). General bilateral amygdalar volume was found to be lower in a large cohort of 86 patients with mixed FND,^4^ while higher left amygdala volumes were found in 48 patients with motor FND.^92^ When looking at subregions of the amygdala, lower right lateral amygdala volume was found in 37 female patients with functional seizures, along with higher right central, medial and left anterior amygdalar volume.^38^ Especially considering the central amygdala as a key region for oxytocinergic receptors for inhibiting a fear response, this specific amygdalar enlargement can be of high relevance.^37^ Further, the inverse correlation of *OXTR* methylation and FC around the amygdala to emotion-related region suggested that the inability of OXT to bind to its receptor sites might affect emotional processing in the brain.^9^ Assuming that higher *OXTR* methylation leads to fewer OXT binding sites, less effective OXT might be available in the system. Eventhough we do not know the exact mechanism behind such effects, different biological routes have been discussed within this oxytocinergic system that might lead to the observed changes of amygdalar volume.^9^ Enhanced *OXTR* methylation as reported here might have a similar effect as low levels of OXT in the periphery to begin with and, therefore, we could interpret that little of these protective effects discussed might be in place for FND.

Further, amygdalar volume has been suggested to be shaped by sex and genetic factors but also to be developed through early social experiences.^93^ Another study discussed the important consideration of differential sex effect when looking at the correlation of OXT in saliva and amygdalar volume, for which a positive correlation was reported for males and a negative correlation was reported for females.^94^ For a disorder that is not equally distributed across sex, such sex-dependent differences in biological mechanisms and interaction seem relevant to consider.

In the FND group, OXT levels correlated negatively with FC from the right amygdala to the hippocampus, the left PCC, the left OFC and the right inferior temporal cortex and positively with amygdalar connectivity to right inferior partietal cortex. Contrarily, in HC only, the OXT levels correlated with both left and right amygdalar FC with regions such as the insula, the thalamus or the superior parietal lobe (Fig. 3). Previous results have shown that OXT would increase effective homotopic interhemispheric connectivity in various brain regions,^95^ especially on effective connections from regions in the salience network and the posterior midline default more network.^95^ Likewise, altered coupling between the salience and default mode networks has been reported in this cohort.^47^ Interestingly, salivary OXT levels have also been associated with lower interregional functional coupling between the amygdala and hippocampus, which could further be reduced by single and also repetitive administration of OXT in males with autism spectrum disorder.^39,68^ However, as we did not find a significant difference in salivary OXT levels between the two groups, we interpret that there might be a distinctive mechanism about the effect of OXT on a neuronal basis for FND patients, eventhough OXT seems similarly available in the periphery. This finding might be explained by the enhanced methylation rates of the *OXTR* that correlated with higher OXT levels in FND, but lower OXT levels in HC. While this may be mediated by the *OXTR* rs53576 genotype, (hyper)methylation can also lead to an atypical development of brain structures after trauma.^96^

### Limitations

A limitation of this study is that—eventhough some studies argue for high stability^97^—large variation in the baseline levels of OXT has been identified even within individuals, thus the use of a single time assessment is not ideal.^56^ The estimation of intra-class correlation coefficient for a single OXT measurement in saliva has been estimated to be 0.23^56^; thus, for more validity, we would have required multiple samples per individual throughout the day. Also related to saliva sample quality, there were no strict instructions for the saliva collection, meaning that other confounding factors such as appetite, satiation, body mass index, exercising or being sexually active on the day of the collection could have influenced the OXT levels of the participants.^98-100^ A major limitation is the overall small sample size with regards to genetic analyses.^46^ The absence of significant association between AA-genotype and OXT levels in HC could be due to the fact that only *N* = 6 HC were AA-carriers. While we state that the AA-genotype might represent a contributing factor for FND, a much larger sample would be required to make a clear statement on the genetic findings. Lastly, our patient cohort has only been compared with HC, which prevents making conclusions on the specificity of the findings to FND in comparison to other psychiatric disorders, especially as the interpretation of results was guided by the knowledge of findings from other clinical population, like from anxiety, autism spectrum or psychosis research. Eventhough we excluded patients with severe psychiatric comorbidities and corrected for anxiety and depression, the lack of systematic psychiatric evaluation does not allow us to fully exclude psychiatric comorbidity (e.g. post-traumatic stress disorder), which is commonly observed in FND, thus, specificity of the findings cannot be entirely controlled for.^101,102^

## Conclusion

Our findings point toward a unique interaction between peripheral OXT and *OXTR* methylation in patients with FND in a genotype-dependent manner. With higher methylation rates of the *OXTR*, patients presented with increased peripheral OXT levels, which might denote a compensatory mechanism during which more OXT is secreted as a consequence of lower availability of the oxytocin receptor binding sites. This might stand in direct relationship to the herein-reported amygdalar volumetric and FC results, suggesting that peripherally patients do not differ from HC, but on a neuronal level, the inability of OXT to bind to its receptor might affect the FC of the amygdala and related regions. In summary, the rs53576 SNP of the *OXTR* gene might represent a genetic contributing factor for FND that further affects *OXTR* methylation, peripheral OXT levels and amygdala structure and connectivity.

## Supplementary Material

fcae455_Supplementary_Data

## Data Availability

The data that support the findings of this study are available from the corresponding author, on request. An explanatory code and all statistical analyses and plots generated can be found on https://github.com/webersamantha/Oxytocin_FND.
