# Supplementary material for: Salivary oxytocin and amygdalar alterations in functional neurological disorders
Source: Brain Commun. 2024 Dec 16;7(1):fcae455. doi: 10.1093/braincomms/fcae455 (PMC11670354; doi:10.1093/braincomms/fcae455)
Supplement: fcae455_Supplementary_Data [file fcae455_supplementary_data.pdf]

Supplementary material

Salivary Oxytocin and Amygdalar Alterations in Functional Neurological Disorders

Samantha Weber[1,2†], Natascha Stoffel[1,3,4†], Juan Ansede-Bermejo[5], Raquel Cruz[5,6,7,8], Álvaro Del Real Bolt[9], Rupert Bruckmaier[10], Ángel Carracedo[5,6,7,8,11], Selma Aybek[3]

[†] **Samantha Weber and Natascha Stoffel contributed equally to this work.**

Author affiliations:

1 Department of Neurology, Psychosomatic Medicine Unit, Inselspital Bern University Hospital, University of Bern, 3012 Bern, Switzerland

2 University of Zurich, Psychiatric University Hospital Zurich, Department of Psychiatry, Psychotherapy and Psychosomatics, 8032 Zurich, Switzerland.

3 Faculty of Science and Medicine, University of Fribourg, 1700 Fribourg, Switzerland

4 Graduate School of Health Science (GHS), University of Bern, 3013 Bern, Switzerland

5 Centro Nacional de Genotipado (CEGEN), Universidade de Santiago de Compostela, Santiago de Compostela, Spain.

6 Centre for Biomedical Network Research on Rare Diseases (CIBERER), Instituto de Salud Carlos III, Madrid, Spain.

7 Instituto de Investigación Sanitaria de Santiago (IDIS), Santiago de Compostela, Spain.

8 Centro Singular de Investigación en Medicina Molecular y Enfermedades Crónicas (CIMUS), Universidade de Santiago de Compostela, Santiago de Compostela, Spain.

9 Medicine and Psychiatry Department. University of Cantabria, Santander, Spain.

10 Veterinary Physiology, Vetsuisse Faculty, University of Bern, 3012 Bern, Switzerland

11 Fundación Pública Galega de Medicina Xenómica, Sistema Galego de Saúde (SERGAS), Santiago de Compostela, Spain.

Covariates of oxytocin

Concerning the methodology of measuring peripheral oxytocin and which variables to include as covariates, or what to control for, unfortunately there is not yet really a standard procedure.[1] Clearly, there are important covariates to be taken into account for an oxytocin correlational analysis, which we also considered for our study and further want to discuss hereby. A metaanalysis suggest that women have higher OXT levels compared to men, older people have higher OXT levels compared to younger people, as well people would have higher OXT levels in the afternoon compared to in the morning.[2]

To cite some of the original articles and our undertaking in this study on that regard:

Women were shown to have higher OXT levels compared to men, when looking at naturally cycling women in their follicular phase.[3] In a study with depressed patients, the difference of OXT levels between depressed and healthy participants was only detectable in females, with a trend that generally males had lower OXT then females,[4] supporting the notion that OXT has a differential effect based on sex.[5] Our study does not control artificially for the variable of sex, as this would not mirror a realistic image of FND,[6] but matches accordingly the patient group with same-sex healthy controls.

Further, levels do fluctuate throughout the female menstrual cycle, leading to higher OXT levels from early follicular phase up until ovulation with significantly lower levels in the mid-luteal phase.[7,8] For women after menopause, OXT levels are lower compared to pre-menopausal women in early follicular phase.[9] OXT levels are also higher for women on hormonal contraception.[10,11] It is thus important to allow such variables to vary accordingly, as also debated by Bale & Epperson (2017).[12] As we here to did not want to control for a specific phase in menstrual cycle, we included the variable menstrual cycle as a numeric variable that allows to represent the dynamic changes most adequately. Also, it seems that also age could play a role, with higher OXT levels in younger compared to older people,[13] whereas the difference in OXT levels could potentially also be explained by age-related skills as socio-emotional capabilities or experiences through life.[14] Here too, we point out that our population with FND has been matched with the appropriate age group in the control population.

Whole-Brain Functional Connectivity

Whole-brain resting-state functional connectivity (FC) was calculated according to standard procedure.[16–19] As such, the functional images were parcellated into the 90 cortical and subcortical regions of interest according to the automatic anatomic labelling atlas (AAL). The region-averaged time courses were extracted, and FC was computed using Pearson's correlation coefficient between the time series of the seed with each of the regions. The correlation coefficients were further z-scored using Fisher z transformation. Significant differences in functional connectivity between patients ($N = 54$) and controls ($N = 64$) were assessed using two-tailed multiple t-tests, corrected using false discovery rate (FDR) at a significance threshold of $P < \alpha$, where alpha level (α) was set to 0.05.

Compared to HC, patients showed increased functional connectivity between the right middle temporal pole and the right gyrus rectus ($t(112) = 4.32$, $P_{FDR} = 0.03$). Moreover, patients showed decreased functional connectivity between the 1) the left insula and the right opercular part of the inferior frontal gyrus ($t(115) = 4.21$, $P_{FDR} = 0.03$), 2) the left insula and the right calcarine gyrus ($t(108) = 4.63$, $P_{FDR} = 0.02$), 3) the left gyrus rectus and the left superior occipital cortex ($t(110) = -4.22$, $P_{FDR} = 0.03$), 4) the right gyrus rectus and the right cuneus ($t(102) = -4-37$, $P_{FDR} = 0.03$), and 5) the right gyrus rectus and the left superior occipital cortex ($t(115) = -4.64$, $P_{FDR} = 0.02$), Supplementary Figure 1.

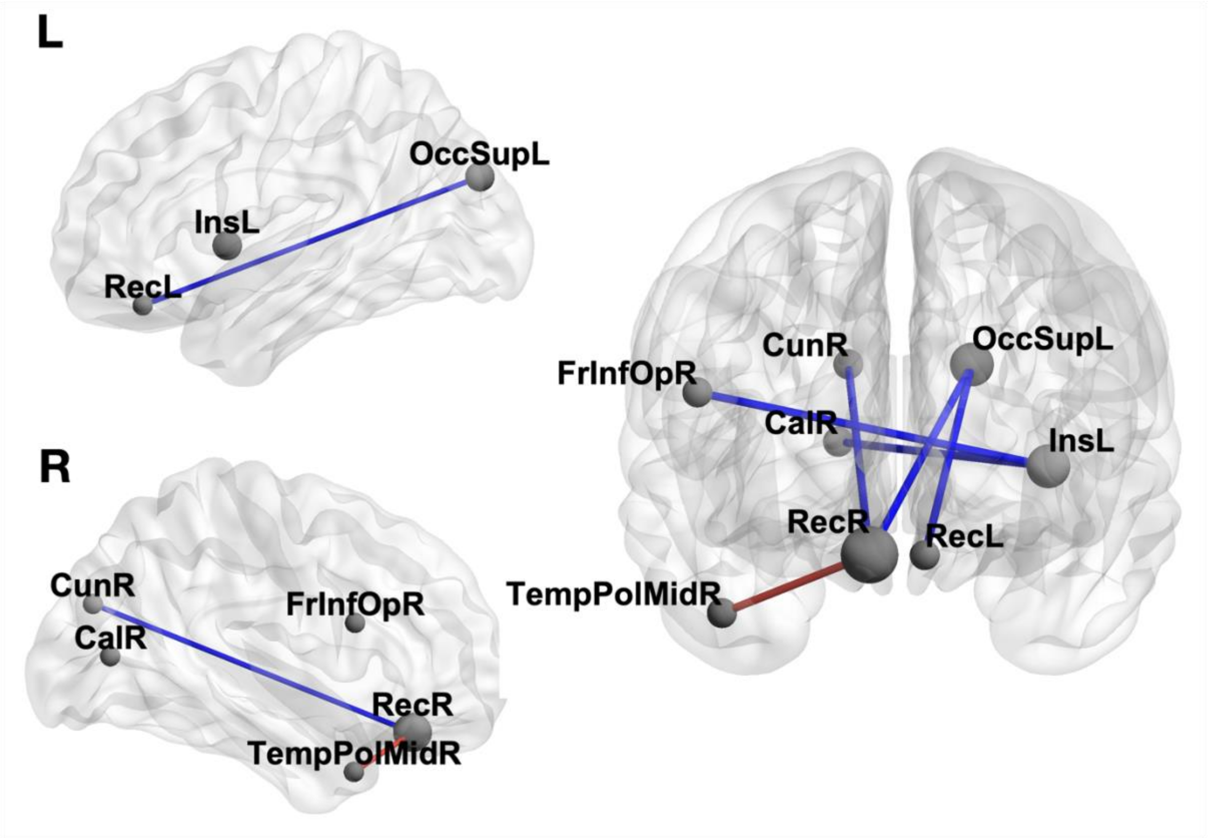

Supplementary Figure 1 Functional connectivity differences between FND patients and healthy controls. Whole-brain resting-state functional connectivity (FC) was calculated by parcellating functional images into 90 cortical and subcortical regions based on the AAL atlas. FC was computed using Pearson's correlation between region-averaged time courses and transformed using Fisher z-scores. Significant differences in functional connectivity between patients ($N = 54$) and controls ($N = 64$) were assessed using two-tailed multiple t-tests, corrected using false discovery rate (FDR) at a significance threshold of $P < \alpha$, where alpha level (α) was set to 0.05. Decreased functional connectivity in patients compared to healthy controls is depicted in blue, while increased functional connectivity is depicted in red. The size of the nodes corresponds to nodal degree. Thickness of the edges correspond to the *P*-value. Abbreviations: RecL: Left gyrus rectus; RecR: Right gyrus rectus: InsL: Left insula; OccSupL: Left superior occipital lobe; CunR: Right Cuneus; CalR: Right Calcarine Gyrus; FrInfOpR: right opercular part of the inferior frontal gyrus; TempPolMidR: right middle temporal pole.

Interaction with Childhood Trauma

There were no significant differences in OXT, *OXTR* methylation or genotype depending on CTQ subscores, Supplementary Figure 2 and Supplementary Figure 3.

Supplementary Figure 2 Interaction of salivary oxytocin and *OXTR* methylation with childhood trauma in FND patients and healthy controls. Scatter plots illustrate the association between different subscales of the Childhood Trauma Questionnaire (CTQ) – Emotional Abuse (Panels A and B), Emotional Neglect (Panels C and D), Physical Neglect (Panel E and F), Physical Abuse (Panel G and H) and Sexual Abuse (Panel I and J) – with *OXTR* methylation (Panels A, C, E, G and I) in *N* = 59 FND patients and *N* = 65 healthy controls. Data points represent individual participants, with their position on the x-axis corresponding to their score on a specific CTQ subscale, and the y-axis corresponding to their *OXTR* methylation level (%) or oxytocin levels (pg/ml). Spearman's rank correlation was used to assess associations between CTQ and biological data. The significance of these correlations was evaluated independently for each group.

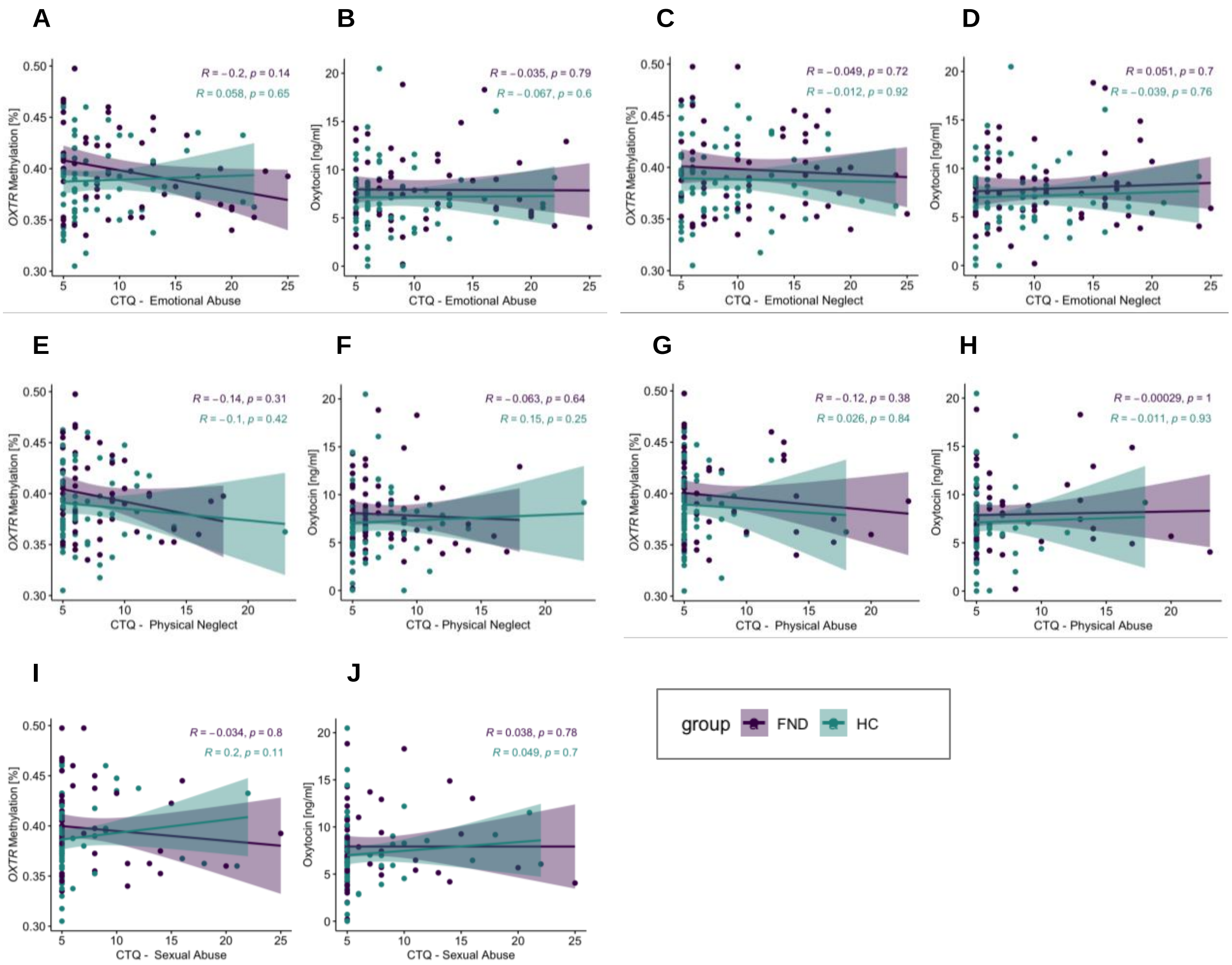

Supplementary Figure 3 Interaction of salivary oxytocin and *OXTR* methylation with childhood trauma stratified according to genotype in FND patients and healthy controls. Scatter plots illustrate the association between different subscales of the Childhood Trauma Questionnaire (CTQ) – Emotional Neglect, Emotional Abuse, Physical Neglect, Physical Abuse and Sexual Abuse– with *OXTR* methylation and oxytocin levels in $N = 59$ FND patients and $N = 65$ healthy controls stratified according to *OXTR* (rs53576) genotype. Data points represent individual participants, with their position on the x-axis corresponding to their score on a specific CTQ subscale, and the y-axis corresponding to their *OXTR* methylation level (%) or oxytocin levels (pg/ml). Spearman's rank correlation was used to assess associations between CTQ and biological data. The significance of these correlations was evaluated independently for each group.

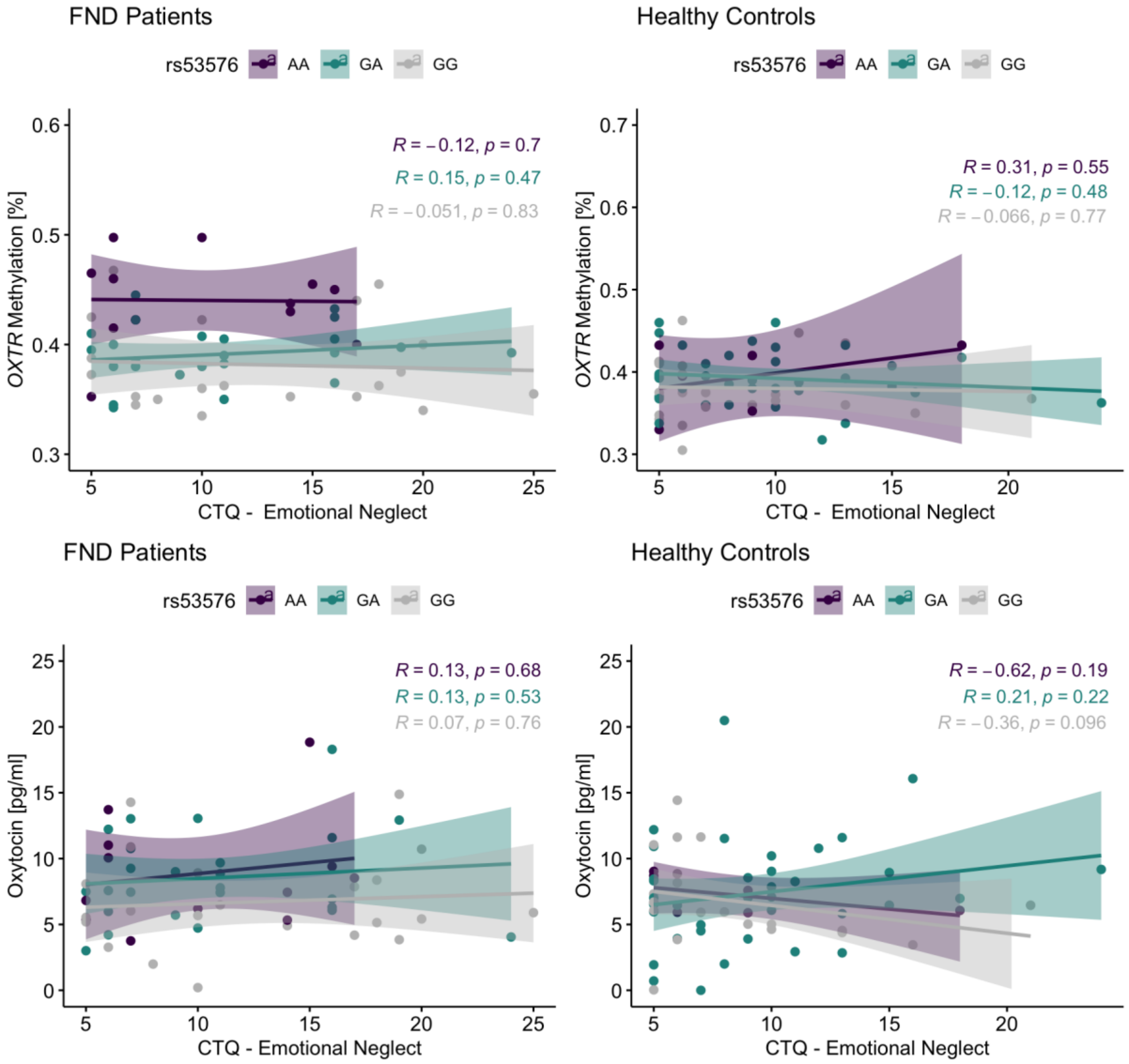

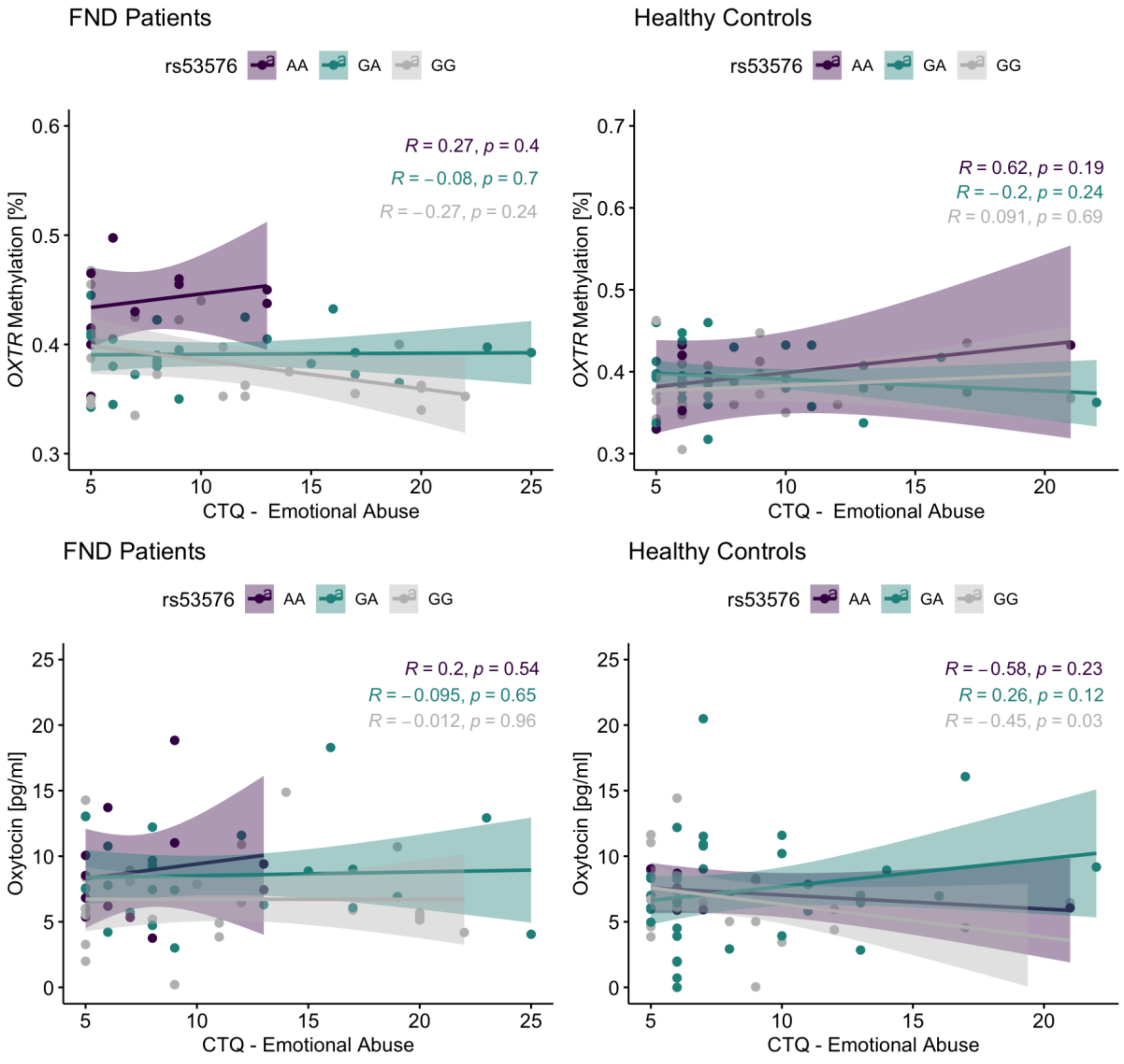
FND Patients
rs53576 AA GA GG
R = 0.27, p = 0.4
R = −0.08, p = 0.7
R = −0.27, p = 0.24
OXTR Methylation [%]
CTQ - Emotional Abuse
Healthy Controls
rs53576 AA GA GG
R = 0.62, p = 0.19
R = −0.2, p = 0.24
R = 0.091, p = 0.69
OXTR Methylation [%]
CTQ - Emotional Abuse
FND Patients
rs53576 AA GA GG
R = 0.2, p = 0.54
R = −0.095, p = 0.65
R = −0.012, p = 0.96
Oxytocin [pg/ml]
CTQ - Emotional Abuse
Healthy Controls
rs53576 AA GA GG
R = −0.58, p = 0.23
R = 0.26, p = 0.12
R = −0.45, p = 0.03
Oxytocin [pg/ml]
CTQ - Emotional Abuse

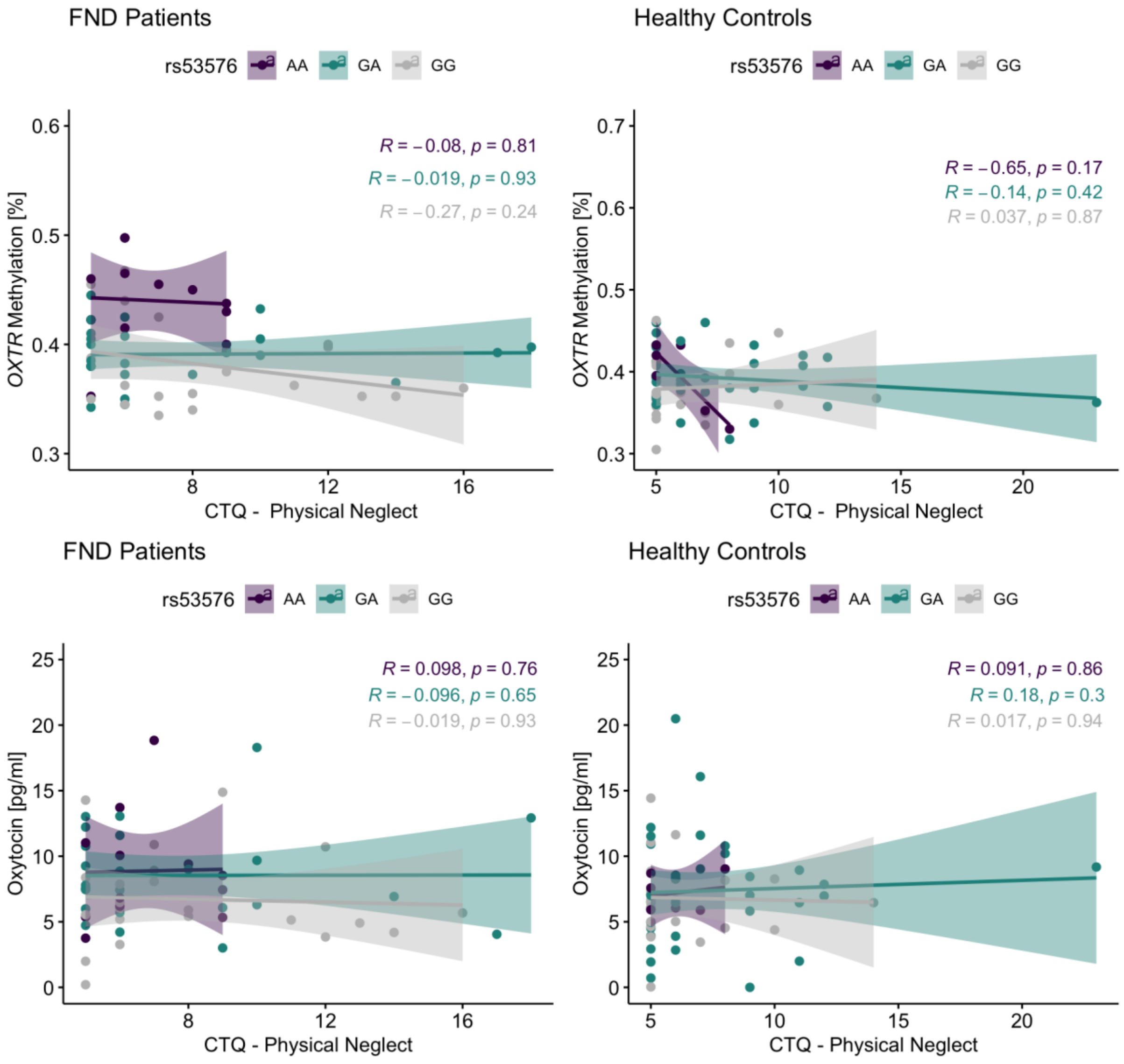

FND Patients
rs53576 AA GA GG
R = –0.08, p = 0.81
R = –0.019, p = 0.93
R = –0.27, p = 0.24
OXTR Methylation [%]
CTQ - Physical Neglect
Healthy Controls
rs53576 AA GA GG
R = –0.65, p = 0.17
R = –0.14, p = 0.42
R = 0.037, p = 0.87
OXTR Methylation [%]
CTQ - Physical Neglect
FND Patients
rs53576 AA GA GG
R = 0.098, p = 0.76
R = –0.096, p = 0.65
R = –0.019, p = 0.93
Oxytocin [pg/ml]
CTQ - Physical Neglect
Healthy Controls
rs53576 AA GA GG
R = 0.091, p = 0.86
R = 0.18, p = 0.3
R = 0.017, p = 0.94
Oxytocin [pg/ml]
CTQ - Physical Neglect

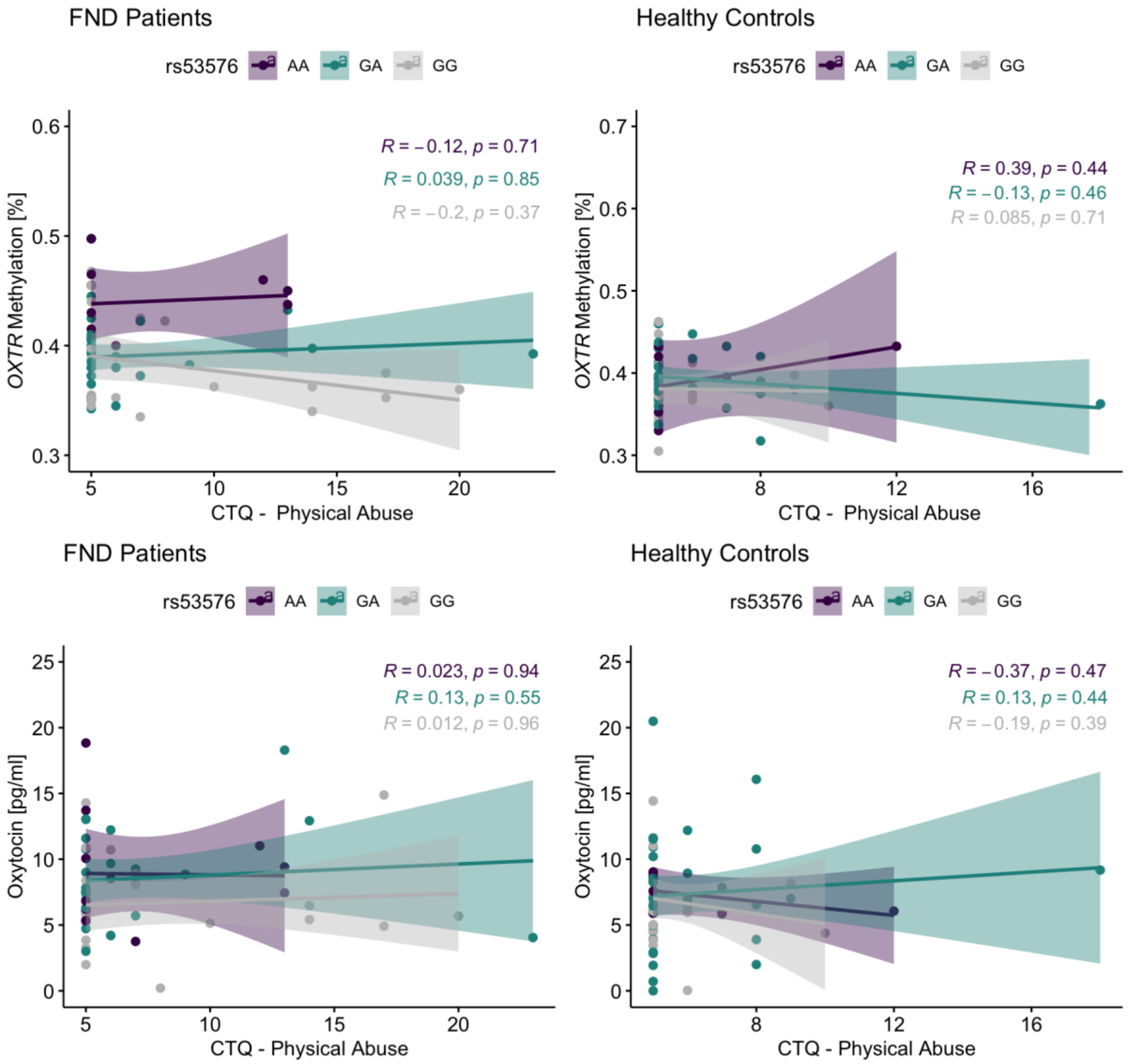
FND Patients
Healthy Controls
rs53576
AA
GA
GG
R = −0.12, p = 0.71
R = 0.039, p = 0.85
R = −0.2, p = 0.37
R = 0.39, p = 0.44
R = −0.13, p = 0.46
R = 0.085, p = 0.71
OXTR Methylation [%]
CTQ - Physical Abuse
R = 0.023, p = 0.94
R = 0.13, p = 0.55
R = 0.012, p = 0.96
R = −0.37, p = 0.47
R = 0.13, p = 0.44
R = −0.19, p = 0.39
Oxytocin [pg/ml]

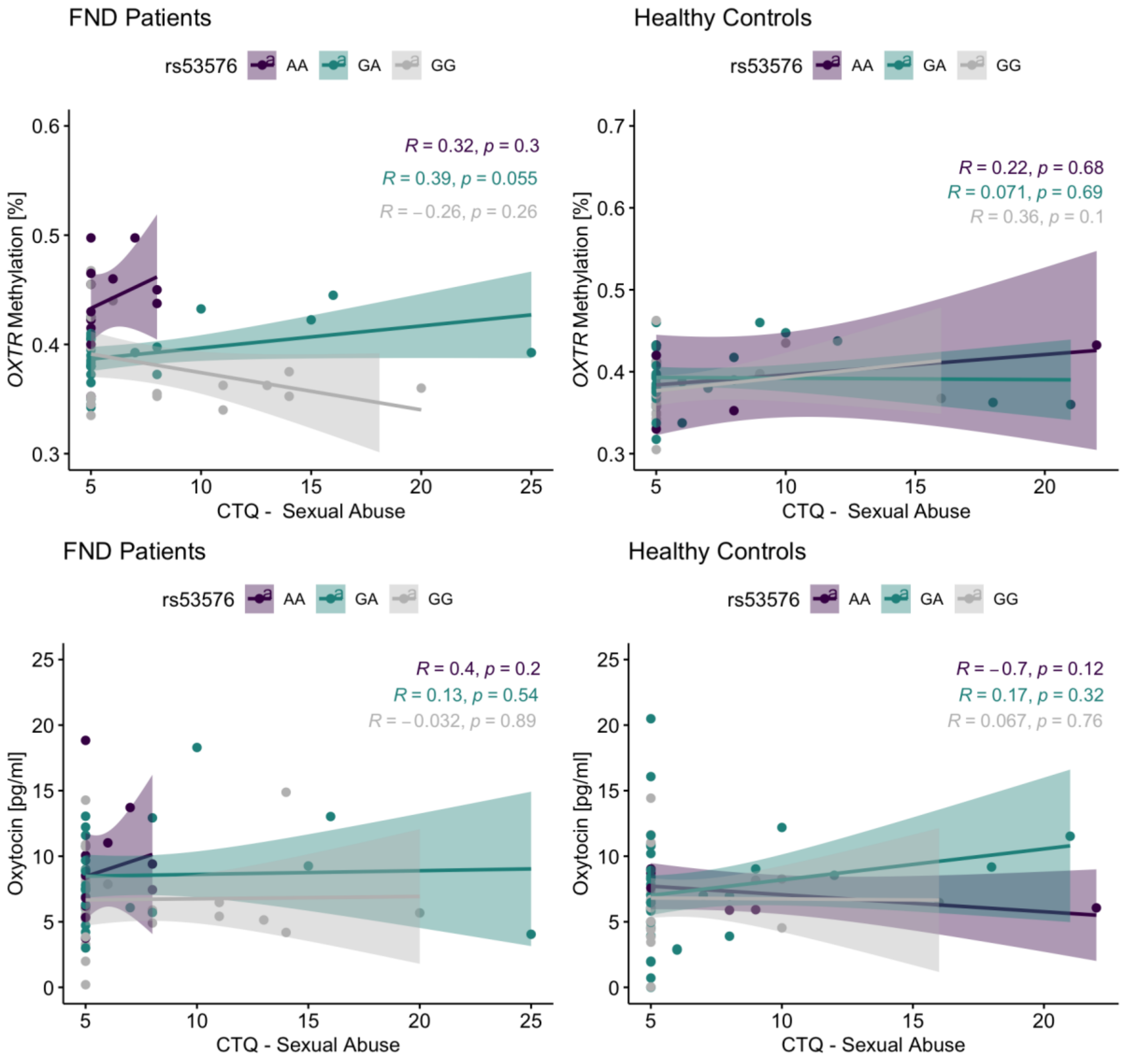
FND Patients
rs53576 AA GA GG
R = 0.32, p = 0.3
R = 0.39, p = 0.055
R = −0.26, p = 0.26
OXTR Methylation [%]
0.3 0.4 0.5 0.6
5 10 15 20 25
CTQ - Sexual Abuse
Healthy Controls
rs53576 AA GA GG
R = 0.22, p = 0.68
R = 0.071, p = 0.69
R = 0.36, p = 0.1
OXTR Methylation [%]
0.3 0.4 0.5 0.6 0.7
5 10 15 20
CTQ - Sexual Abuse
FND Patients
rs53576 AA GA GG
R = 0.4, p = 0.2
R = 0.13, p = 0.54
R = −0.032, p = 0.89
Oxytocin [pg/ml]
0 5 10 15 20 25
5 10 15 20 25
CTQ - Sexual Abuse
Healthy Controls
rs53576 AA GA GG
R = −0.7, p = 0.12
R = 0.17, p = 0.32
R = 0.067, p = 0.76
Oxytocin [pg/ml]
0 5 10 15 20 25
5 10 15 20
CTQ - Sexual Abuse

Interaction with Sex

There was no significant group x sex interaction effect on *OXTR* methylation.

Supplementary Figure 4 *OXTR* methylation in FND patients and healthy controls stratified according to sex. Violin plot representing distribution of raw *OXTR* methylation levels in **(A)** patients with FND (N = 59) and **(B)** healthy controls (N = 65), stratified according to sex. Boxplots indicate median and interquartile range. Using an ANOVA on the fitted data using a linear model, a significant main effect of sex on *OXTR* methylation has been found in HC ($F(1,55) = 6.47$, $P = 0.01$, $d = 0.461$), but not in FND patients.

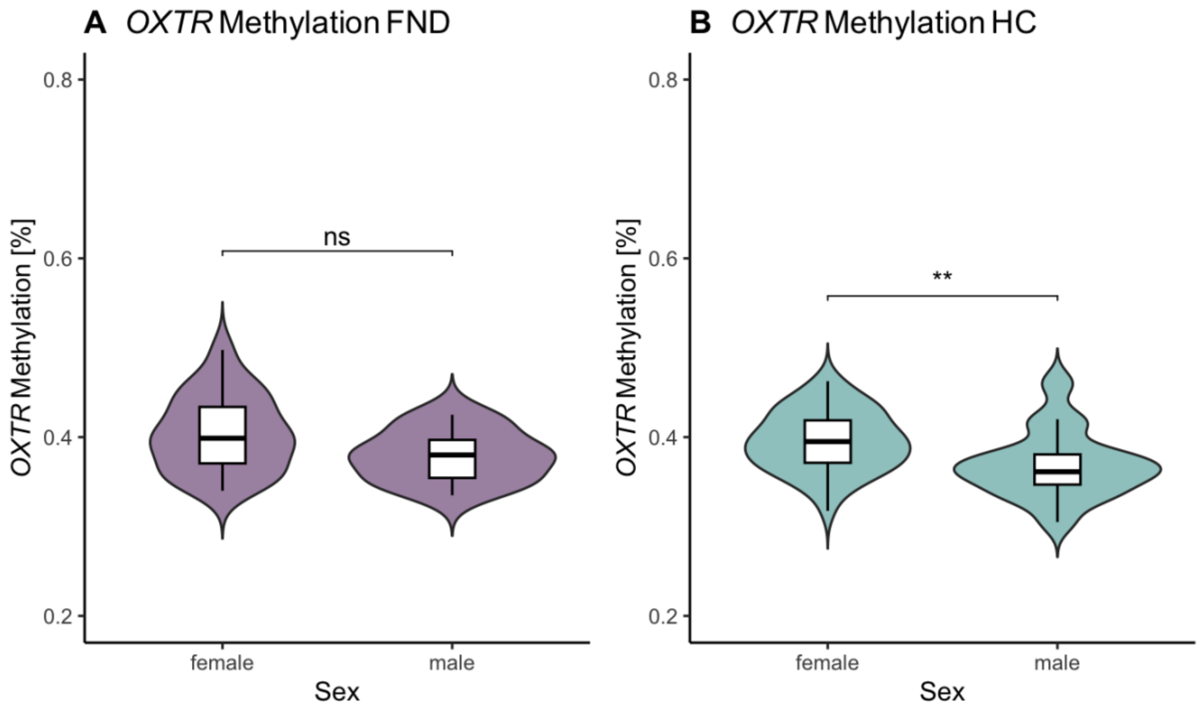

References

1. Tabak BA, Leng G, Szeto A, et al. Advances in human oxytocin measurement: challenges and proposed solutions. *Mol Psychiatry*. Published online August 23, 2022:1-14. doi:10.1038/s41380-022-01719-z

2. Engel S, Laufer S, Miller R, Niemeyer H, Knaevelsrud C, Schumacher S. Demographic, sampling- and assay-related confounders of endogenous oxytocin concentrations: A systematic review and meta-analysis. *Front Neuroendocrinol*. 2019;54:100775. doi:10.1016/j.yfrne.2019.100775

3. Marazziti D, Baroni S, Mucci F, et al. Sex-Related Differences in Plasma Oxytocin Levels in Humans. *Clin Pract Epidemiol Ment Health*. 2019;15:58-63.

4. Ozsoy S, Esel E, Kula M. Serum oxytocin levels in patients with depression and the effects of gender and antidepressant treatment. *Psychiatry Res*. 2009;169(3):249-252. doi:10.1016/j.psychres.2008.06.034

5. Grace SA, Rossell SL, Heinrichs M, Kordsachia C, Labuschagne I. Oxytocin and brain activity in humans: A systematic review and coordinate-based meta-analysis of functional MRI studies. *Psychoneuroendocrinology*. 2018;96:6-24. doi:10.1016/j.psyneuen.2018.05.031

6. McLoughlin C, Hoeritzauer I, Cabreira V, et al. Functional neurological disorder is a feminist issue. *J Neurol Neurosurg Psychiatry*. 2023;94(10):855-862. doi:10.1136/jnnp-2022-330192

7. Engel S, Klusmann H, Ditzen B, Knaevelsrud C, Schumacher S. Menstrual cycle-related fluctuations in oxytocin concentrations: A systematic review and meta-analysis. *Front Neuroendocrinol*. 2019;52:144-155. doi:10.1016/j.yfrne.2018.11.002

8. Salonia A, Nappi RE, Pontillo M, et al. Menstrual cycle-related changes in plasma oxytocin are relevant to normal sexual function in healthy women. *Horm Behav*. 2005;47(2):164-169. doi:10.1016/j.yhbeh.2004.10.002

9. Maestrini S, Mele C, Mai S, et al. Plasma Oxytocin Concentration in Pre- and Postmenopausal Women: Its Relationship with Obesity, Body Composition and Metabolic Variables. *Obes Facts*. 2018;11(5):429-439. doi:10.1159/000492001

10. Silber M, Almkvist O, Larsson B, Stock S, Uvnäs-Moberg K. The effect of oral contraceptive pills on levels of oxytocin in plasma and on cognitive functions. *Contraception*. 1987;36(6):641-650. doi:10.1016/0010-7824(87)90037-0

11. Stock S, Silber M, Uvnäs-Moberg K. Elevated Plasma Levels Of Oxytocin In Women Taking Low-Dose Oral Contraceptives: Identification Of The Plasma Oxytocin With High Performance Liquid Chromatography. *Acta Obstet Gynecol Scand*. 1989;68(1):75-78. doi:10.3109/00016348909087694

12. Bale TL, Epperson CN. Sex as a Biological Variable: Who, What, When, Why, and How. *Neuropsychopharmacology*. 2017;42(2):386-396. doi:10.1038/npp.2016.215

13. Aulinas A, Pulumo RL, Asanza E, et al. Endogenous Oxytocin Levels in Relation to Food Intake, Menstrual Phase, and Age in Females. *J Clin Endocrinol Metab*. 2018;104(4):1348-1356. doi:10.1210/jc.2018-02036

14. Ebner N, Maura G, Macdonald K, Westberg L, Fischer H. Oxytocin and socioemotional aging: Current knowledge and future trends. *Front Hum Neurosci*. 2013;7. Accessed April 12, 2023. https://www.frontiersin.org/articles/10.3389/fnhum.2013.00487

15. Dadds MR, Moul C, Cauchi A, et al. Methylation of the oxytocin receptor gene and oxytocin blood levels in the development of psychopathy. *Dev Psychopathol*. 2014;26(1):33-40. doi:10.1017/S0954579413000497

16. Richiardi J, Eryilmaz H, Schwartz S, Vuilleumier P, Van De Ville D. Decoding brain states from fMRI connectivity graphs. *NeuroImage*. 2011;56(2):616-626. doi:10.1016/j.neuroimage.2010.05.081

17. Richiardi J, Gschwind M, Simioni S, et al. Classifying minimally disabled multiple sclerosis patients from resting state functional connectivity. *NeuroImage*. 2012;62(3):2021-2033. doi:10.1016/j.neuroimage.2012.05.078

18. Richiardi J, Van De Ville D, Riesen K, Bunke H. Vector Space Embedding of Undirected Graphs with Fixed-cardinality Vertex Sequences for Classification. In: *2010 20th International Conference on Pattern Recognition*. IEEE; 2010:902-905. doi:10.1109/ICPR.2010.227

19. Weber S, Heim S, Richiardi J, et al. Multi-centre classification of functional neurological disorders based on resting-state functional connectivity. *NeuroImage Clin*. 2022;35:103090. doi:10.1016/j.nicl.2022.103090
